# Athletic bioimpedance-based equations underestimate fat free mass components in male elite soccer players: development and validation of new soccer-specific predictive models

**DOI:** 10.1186/s12967-023-04795-z

**Published:** 2023-12-15

**Authors:** Francesco Campa, Tindaro Bongiovanni, Alessio Rossi, Giuseppe Cerullo, Andrea Casolo, Giulia Martera, Athos Trecroci, Tatiana Moro, Antonio Paoli

**Affiliations:** 1 Department of Biomedical Sciences, University of Padua, 35131 Padua, Italy; 2 Department of Biomedical and Neuromotor Sciences, University of Bologna, Bologna, Italy; 3 Department of Performance, Palermo Football Club, Palermo, Italy; 4 Computer Science, University of Pisa, Pisa, Italy and National Research Council (CNR), Institute of Information Science and Technologies (ISTI), Pisa, Italy; 5 Department of Performance Nutrition, Spezia Calcio, La Spezia, Italy; 6 Department of Biomedical Science for Health, University of Milan, Milan, Italy

**Keywords:** Bioelectric impedance analysis, BIA, BIVA, Body composition, Lean soft tissue, Somatotype

## Abstract

**Background:**

Bioelectrical impedance analysis (BIA) is a rapid and user-friendly technique for assessing body composition in sports. Currently, no sport-specific predictive equations are available, and the utilization of generalized formulas can introduce systematic bias. The objectives of this study were as follows: (i) to develop and validate new predictive models for estimating fat-free mass (FFM) components in male elite soccer players; (ii) to evaluate the accuracy of existing predictive equations.

**Methods:**

A total of 102 male elite soccer players (mean age 24.7 ± 5.7 years), participating in the Italian first league, underwent assessments during the first half of the in-season period and were randomly divided into development and validation groups. Bioelectrical resistance (R) and reactance (Xc), representing the bioimpedance components, were measured using a foot-to-hand BIA device at a single frequency of 50 kHz. Dual-energy X-ray absorptiometry was employed to acquire reference data for FFM, lean soft tissue (LST), and appendicular lean soft tissue (ALST). The validation of the newly developed predictive equations was conducted through regression analysis, Bland–Altman tests, and the area under the curves (AUC) of regression receiver operating characteristic (RROC) curves.

**Results:**

Developed models were: FFM = − 7.729 + (body mass × 0.686) + (stature^2^/R × 0.227) + (Xc × 0.086) + (age × 0.058), R^2^ = 0.97, Standard error of estimation (SEE) = 1.0 kg; LST = − 8.929 + (body mass × 0.635) + (stature^2^/R × 0.244) + (Xc × 0.093) + (age × 0.048), R^2^ = 0.96, SEE = 0.9 kg; ALST = − 24.068 + (body mass × 0.347) + (stature^2^/R × 0.308) + (Xc × 0.152), R^2^ = 0.88, SEE = 1.4 kg. Train-test validation, performed on the validation group, revealed that generalized formulas for athletes underestimated all the predicted FFM components (p < 0.01), while the new predictive models showed no mean bias (p > 0.05), with R^2^ values ranging from 0.83 to 0.91, and no trend (p > 0.05). The AUC scores of the RROC curves indicated an accuracy of 0.92, 0.92, and 0.74 for FFM, LST, and ALST, respectively.

**Conclusions:**

The utilization of generalized predictive equations leads to an underestimation of FFM and ALST in elite soccer players. The newly developed soccer-specific formulas enable valid estimations of body composition while preserving the portability of a field-based method.

## Introduction

Body composition evaluation is a common practice in soccer. By measuring body mass components medical staff and coaches can assess nutritional status, energy expenditure, personalize a dietary intervention, and monitor the response of training regimens or detraining periods across the competitive season [1, 2]. Although the simplest way to analyse body composition is to consider body mass as the sum of fat mass and fat-free mass (FFM), the quantification of smallest FFM components allows for a higher accuracy through a compartmental-based assessment [3]. Indeed, FFM can be split into several components according to a molecular (e.g., total body water and bone mineral content), cellular (e.g., intra- and extracellular water, and body cell mass), or tissue (e.g., skeletal muscle, whole-body and appendicular lean soft tissue) organization [4].

Among the most accurate techniques for evaluating body composition, the dual-energy X-ray absorptiometry (DXA) is considered a reference method to obtain total and segmental estimates of FFM components, such as lean soft tissue (LST) and appendicular lean soft tissue (ALST). However, since its application is limited due to high costs and radiation exposure, alternative methods such as the bioelectrical impedance analysis (BIA) are often utilized for routine assessments [5]. Based on four different technologies (i.e., hand to hand, leg to leg, foot to hand, and segmental), many BIA devices have been developed through the years, working at a wide range of sampling frequencies [6]. In addition, the bioelectrical impedance spectroscopy (BIS), which represents a BIA modality, uses a wider range of frequencies to derive the bioelectrical measures, involving them in nonlinear mathematical models to estimate intra- and extracellular resistance values, possibly countering some of the limitations relate to the use of BIA-based predictive models [7]. With BIA it is possible to assess the body impedance which is composed of the bioelectrical resistance (R) and reactance (Xc) [impedance = (R^2^ + Xc^2^)^0.5^] [8]. Bioelectrical R represents the opposition offered by the body to the flow of an alternating electrical current through the conductive volume and is inversely related to the water and electrolyte content of tissues [8]. Bioelectrical Xc, which is detectable by phase-sensitive BIA devices only, is related to the capacitance properties of the cell membrane and to variations that can occur depending on its integrity, function, and sampling frequency [8]. Starting from the relationships between bioelectrical properties and FFM components [9, 10], it has been possible to implement a wide range of BIA-based predictive models over the years [6].

The BIA-based predictive equations use different variables such as stature, body mass, age, and bioelectrical parameters to estimate the amounts of different body components. These equations are developed using BIA data collected from a specific population and reference measurements obtained with more accurate methods (e.g., DXA). Regression models are then created to establish relationships between the bioimpedance and the reference measurements. Once developed, these formulas can be applied to individuals within the same population to estimate body composition parameters without the need for more invasive or time-consuming methods [11]. Nowadays, BIA is widely used in soccer to personalize dietary intervention by calculating the necessary protein intake [12] starting from the FFM or using it for evaluating the energy expenditure [13]. In addition, since LST and ALST refer to muscles and their associate structures [14], their assessment is particularly informative in soccer players, given its relation to strength and power expressions [15, 16]. Furthermore, BIA-based estimation of FFM allows derivation of the fat mass as the difference between body mass and FFM in accordance with the two-component model of body composition [17]. Particularly, assessing the fat mass during the preparatory phase it is common practice in soccer, since an excess of body fat can compromise aerobic capacity and agility during repeated sprints [18, 19]. For all the aforementioned reasons, the use of BIA has become popular in the context of sport, representing a low-cost and user-friendly tool for assessing body composition [5].

Recent studies showed that it is crucial to use BIA-based equations that have been developed and validated in a similar population as the one being assessed [20, 21]. Particularly, the use of predictive equations developed in the general population results in an underestimation of FFM components when applied to athletes [22]. This loss of accuracy may happen when anthropometric characteristics differ among the subjects involved in the development studies and the subjects on which these formulas are applied [11]. In terms of body composition, each sport may have peculiar features as result of different game demands [14, 23]. For example, soccer players are on average shorter and lighter than volleyball or basketball players, resulting in a lower FFM as well as a different morphology [24, 25]. In fact, tall stature does not always represent a determining factor in soccer, unlike in volleyball or basketball where the ball must be pushed over a net or into a basket, respectively. Considering the influence of FFM components on physical performance, soccer players may be impacted by a high legs LST since they must repeatedly lift their body mass against gravity to perform high-speed sprints with changes of direction [15, 26]. As result of these peculiarities, recent studies [24, 27] have shown that soccer players present different bioelectrical characteristics compared to other athletes [28] and this could make it necessary to develop dedicated predictive equations. Purposely, bioelectrical references for soccer players were provided through the years [29, 30]. However, such as references refer to a qualitative BIA-based approach called bioelectrical impedance vector analysis (BIVA), which by the raw bioelectrical proprieties can be simultaneously assessed as a vector among a graph and compared with population-specific tolerance ellipses [5, 31].

To date, only BIA-based predictive equations developed that include athletes of different sports are available in literature [32, 33] and no other study has validated BIA prediction equations for estimating FFM components in soccer players. As aforementioned, since each sport implies different bioelectrical and body composition characteristics [14, 18], our hypothesis was that the use of sport-specific instead of generalized equations would result in more accurate estimations of FFM, LST, and ALST in elite soccer players. Therefore, the aim of this investigation was to develop new soccer-specific predictive equations and testing their accuracy alongside the already published more generic equations developed on mixed samples of athletes from different sport disciplines.

## Methods

### Participants and study design

A total of 102 male soccer players (age 24.7 ± 5.7 yrs) from the first Italian division (Serie A) were included in this cross-sectional study. Given that a sample size of 77 participants was calculated considering a type 1 error of 5% and a power of 80%, our sample size was sufficient for assuring an adequate power analysis in models development. The inclusion criteria were: (i) ≥ 18 years old; (ii) free from performance-enhancing drugs specifically and any medication in general; (iii) free from the consumption of alcohol and caffeinated beverages for at least 15 h prior to testing. Injured soccer players or with less of 10 h of training per week were excluded. Data collection was carried out during the first half of their competitive season, with assessment procedures conducted early in the morning (from 9 to 11 AM). Informed written consent was obtained from all participants and ethical approval was obtained by Ethic Committee of the local University (approval number 1052019), attesting to the fulfilment of all human research standards set out by the declaration of Helsinki.

### Procedures

Body mass and height were measured to the nearest 0.1 kg and 0.1 cm, respectively, using a scale with stadiometer (Seca, Hamburg, Germany). Body mass index (BMI) was calculated as body mass (kg) divided by squared stature (m^2^). Somatotype components were calculated according to the Heath and Carter method [34], as described elsewhere [25].

Foot-to-hand BIA was performed using a single frequency of 50 kHz device (BIA 101 BIVA^®^PRO, Akern Systems, Firenze, Italy). The participants were instructed to remove all objects containing metal and to stay in a supine position during the measurements, isolated from the ground and electrical conductors, with legs abducted at 45°, shoulders abducted at 30° relative to the body midline, and hands pronated (Campa, Toselli, et al., [17]). After cleaning the skin with isopropyl alcohol, two adhesive electrodes (Biatrodes Akern Srl, Firenze, Italy) were applied on the surface of the right hand and two on the right foot, according to the guidelines for athletes [17, 35]. The precision of the bioelectrical device was assessed before each test session; the test– retest coefficient of variation (CV% = standard deviation/mean × 100%) on duplicate measurements of R and Xc was 0.3% and 0.9%, respectively. Urine-specific gravity has been determined to assure that participants were in a hydrated state, defined as the value of urine-specific gravity ≤ 1.020 [36]. Specific gravity of the first morning urine was determined within 30 min of collection using a clinical hand-held refractometer (ATAGO Co., Tokyo, Japan). Generalized bioimpedance-based models for FFM [33] and ALST [32] estimations were included in the train-test validation analyses. BIA-based predictions of FFM were used to derive the fat mass percentage according to a two-compartmental model, which by fat mass results as the difference between body mass and FFM [17]. To better describe the body composition features of the participants, bioelectrical R and Xc were standardized for the participants’ stature and plotted as a vector within the R-Xc graph according to BIVA. The use of BIVA allowed to compare the bioelectrical characteristics among the development and validation groups and with respect to the soccer reference population [29].

Participants underwent a whole-body DXA on a Lunar Prodigy scan (General Electric, Boston, MA, USA) and the Lunar software (Encore 2003 Version 157 7.0) used for body composition assessment. The scanner was calibrated daily prior to scanning, according to manufacturer indications using a calibration block. DXA was performed to derive whole-body measures of FFM, fat mass, LST, and ALST.

### Statistical analysis

IBM SPSS Statistics version 24.0 (IBM, Chicago, Illinois, USA), BIVA software (Piccoli and Pastori, 2002), and MedCalc Statistical Software v.11.1.1.0, 2009 (Mariakerke, Belgium) were used to analyze the data. All variables were checked for normality, using Kolmogorov–Smirnov test. A train-test validation approach was used to evaluate the predictive goodness of the models developed in this study. 2/3 of the participants were randomly assigned (using random.org) to the train set, while the remaining participants (1/3) to the test group. Descriptive characteristics for the development and validation groups are presented as means ± SD. The independent sample Student’s t test was used to assess differences in general characteristics among the development and validation groups. The two-sample Hotelling’s T^2^ test, which represents a multivariate extension of the independent sample Student’s t test, was preformed to compare the mean impedance vector among the two groups; separate 95% confidence ellipses indicate a difference in bioelectrical proprieties. The ability of the following variables (age, body mass, stature, somatotype, R, and Xc) to predict FFM, LST, and ALST in the development group was assessed using backward stepwise linear regression analysis. During model development, normality of residuals and homogeneity of variance were tested. If more than one variable remained in the model, and to assess multicollinearity, a variance inflation factor (VIF) was calculated for each independent variable. No interactions were found between independent variables; therefore, we used the whole sample in the model development. To cross-validate the developed and the already existing models, the predictive equations were then applied in the test set. A paired sample t-test was employed to compare the mean values obtained from the reference technique and from the BIA-based equations. To assess the accuracy of BIA-based models, validation parameters included the analysis of the coefficient of determination and the pure error. Using Lin’s approach [37] the concordance correlation coefficient (CCC) was calculated and interpreted as suggested by McBride [38] (almost perfect > 0.99; substantial > 0.95–0.99; moderate = 0.90–0.95; and poor < 0.90). The CCC includes measures of precision and accuracy (ρ and Cb, respectively). Agreement between BIA-based models and the criterion procedure was determined using the Bland–Altman method [39], including the analysis of the correlation between the mean and the difference of the methods and an estimate of the 95% limits of agreement (LoA). P < 0.05 was established as the statistical significance for all tests. The Area Under the Curves (AUC) of Regression Receiver Operating Characteristic (RROC) curve [40] to understand the validity of the new formulas. In general, an AUC of lower than 0.7 suggests no discrimination, from 0.7 to 0.8 is considered acceptable, from 0.8 to 0.9 is considered excellent, and more than 0.9 is considered outstanding [41].

## Results

**Fig. 1:**
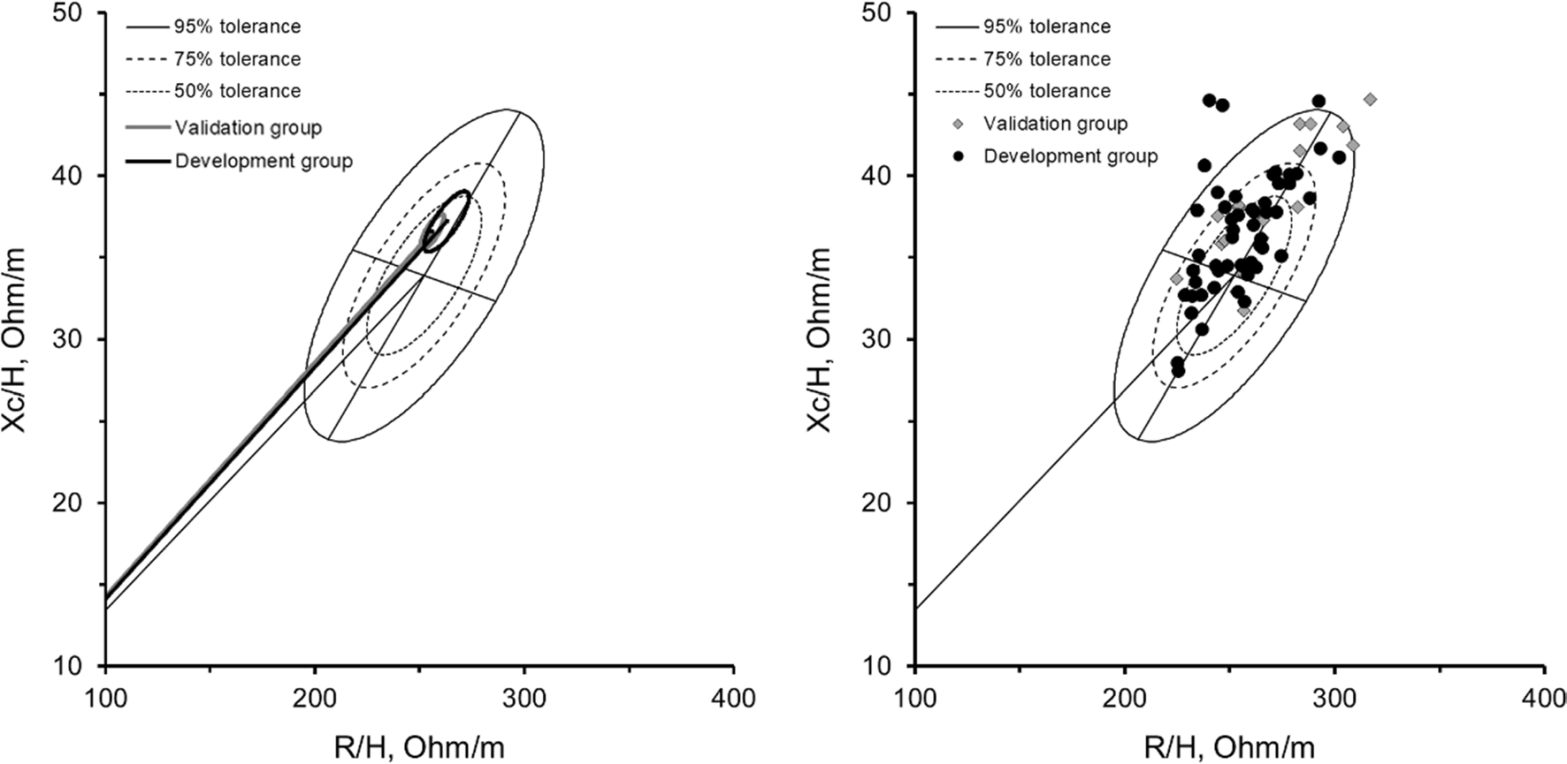
On the left side, mean impedance vectors with the 95% confidence ellipses for the development and validation groups, plotted on the reference tolerance ellipses of the elite soccer players [29]; the two-sample Hotelling’s T test results are included. On the right side, individual vectors for the development and validation groups plotted on the reference ellipses for the elite soccer population are shown

Table 1 and Fig. 1 present the general characteristics for the developmental and validation samples, with no differences observed for the bioelectrical features between the two groups (T = 3.2, F = 1.6, P = 0.207, Mahalanobis distance = 0.39). The mean vectors with their 95% confidence ellipses fell within the 50% tolerance ellipses of the reference male elite soccer population (Fig. 1). Three categories of somatotype resulted from the anthropometric assessment and were balanced mesomorph, ectomorphic mesomorph, and mesomorph-ectomorph.

**Table 1:** Descriptive characteristics and body composition of development and validation groups (mean ± standard deviation)

	Development group (N = 73)	Validation group (N = 29)
Age (yrs)	25.2 ± 5.2	25.9 ± 6.7
Body mass (kg)	80.3 ± 6.3	82.8 ± 5.9
Stature (cm)	184.8 ± 5.8	184.9 ± 4.8
Body mass index (kg/m^2^)	23.6 ± 1.0	24.1 ± 1.2
Endomorphy	1.7 ± 0.3	2.0 ± 0.4
Mesomorphy	4.6 ± 0.6	4.5 ± 0.8
Ectomorphy	2.7 ± 0.5	2.5 ± 0.6
Fat mass (kg)	9.4 ± 1.6	10.2 ± 2.0
Fat mass (%)	11.6 ± 1.6	12.3 ± 2.3
Fat-free mass (kg)	71.5 ± 5.6	72.6 ± 5.8
Lean soft tissue (kg)	67.6 ± 5.3	68.7 ± 5.4
Appendicular lean soft tissue (kg)	36.6 ± 3.9	37.3 ± 3.3
Resistance (ohm)	471.5 ± 29.4	484.4 ± 35.8
Reactance (ohm)	67.4 ± 5.2	68.7 ± 5.9
Phase angle (degree)	8.2 ± 0.7	8.1 ± 0.7

Table 2 shows the new BIA-based predictive equations obtained on the development group. Different models resulted from the development analysis for FFM, LST, and ALST. Only significant variables contributing to the estimates using backward stepwise approach were included in the models. Developed models were:

1. FFM = − 7.729 + (body mass × 0.686) + (stature^2^/R × 0.227) + (Xc × 0.086) + (age × 0.058), R^2^ = 0.97, SEE = 1.0 kg;
2. LST = − 8.929 + (body mass × 0.635) + (stature^2^/R × 0.244) + (Xc × 0.093) + (age × 0.048), R^2^ = 0.96, SEE = 0.9 kg;
3. ALST = − 24.068 + (body mass × 0.347) + (stature^2^/R × 0.308) + (Xc × 0.152), R^2^ = 0.88, SEE = 1.4 kg.

**Table 2:** Developed bioelectrical impedance models for fat-free mass, total, and appendicular lean soft tissue predictions

	Unstandardized coefficient β	Standardized coefficient β	R^2^	SEE (kg)	VIF
Fat-free mass (kg)			0.97	1.04	
Intercept	− 7.729				
BM (kg)	0.686	0.774			1.48
S^2^/R (cm^2^/Ω)	0.227	0.268			3.98
Xc (Ω)	0.086	0.080			1.56
Age (years)	0.058	0.054			1.09
Lean soft tissue (kg)			0.96	0.99	
Intercept	− 8.929				
BM (kg)	0.635	0.751			3.42
S^2^/R (cm^2^/Ω)	0.244	0.301			3.99
Xc (Ω)	0.093	0.091			1.52
Age (years)	0.048	0.046			1.00
Appendicular lean soft tissue (kg)			0.88	1.35	
Intercept	− 24.068				
BM (kg)	0.347	0.561			3.46
S^2^/R (cm^2^/Ω)	0.308	0.521			3.98
Xc (Ω)	0.152	0.203			1.52

BM, body mass; S, stature (cm); R, resistance (Ω); Xc, reactance (Ω); R^2^, coefficient of determination; SEE, standard error of the estimate; VIF, variation inflation factor

**Fig. 2:**
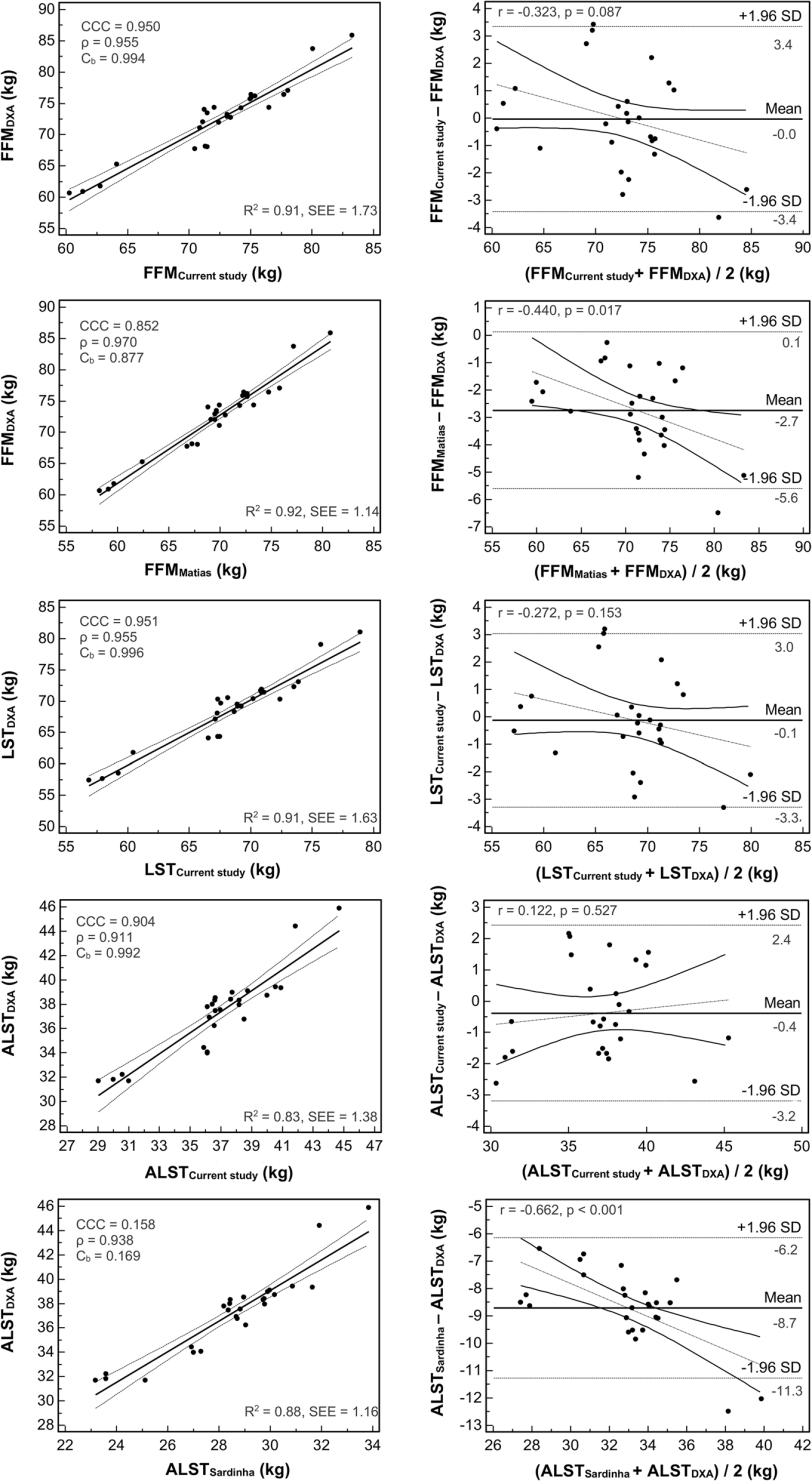
On the left side the scatterplots with the relationship between the predicted and the reference data. On the right side the results of Bland–Altman analyses

A train-test validation was performed and the results of the regression parameters, CCC, and agreement analyses are presented in Fig. 2. No differences between methods were observed for the newly developed sport-specific equations (P > 0.01), whereas routinely used generic athletic equations underestimated (P < 0.01) FFM and ALST (Fig. 2). Concerning the new soccer-specific models there were no significant association between the differences and the means of the BIA- and DXA-derived variables, while the existing athletic predictive equations showed significant and negative trends for all the predicted values (Fig. 2). No mean differences between the predicted and reference percentage of fat mass (12.3% ± 0.8; P = 0.954) were observed when the new soccer-specific model was used for assessing FFM. On the contrary, the use of FFM derived by the athletic generalized equation [33] resulted in an overestimation (15.6% ± 1.3; P < 0.001) of the percentage of fat mass.

**Fig. 3:**
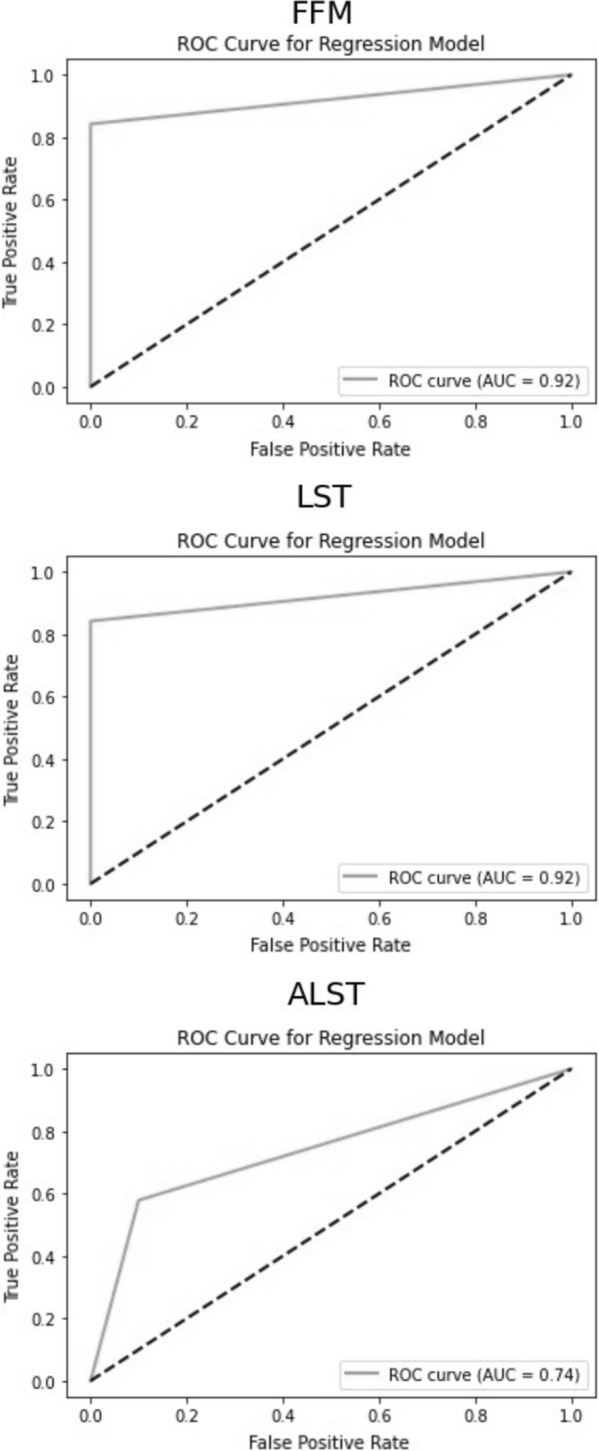
Area Under the Curves (AUC) of Regression Receiver Operating Characteristic (RROC) curves for fat-free mass (FFM), lean soft tissue (LST), and appendicular lean soft tissue (ALST)

AUC score of RROC shows an outstanding accuracy of FFM (0.92) and LST (0.92) formulas, while an acceptable one for ALST (0.74), as presented in Fig. 3. Based on the AUC results all the formulas could be considered valid for the prediction purpose.

## Discussion

This investigation began from the hypothesis that the use of generalized BIA-based equations would have resulted in less accuracy than sport-specific equations when applied in a specific context of sport. For this reason, we recruited a group of elite soccer players with the aim to develop and validate new predictive equations, while testing the performance of already published generalized formulas for athletes. The use of generalized predictive equations resulted in an underestimation of FFM and ALST in the soccer players. In contrast, the new soccer-specific formulas provided more accurate estimations of body composition. The new predictive equations presented in this study allows, for the first time, to obtain valid estimation of body composition using BIA-based procedures specifically developed for elite soccer players.

Three series of predictive models were developed for estimating FFM, LST, and ALST from bioelectrical (R and Xc), anthropometric measures (body mass and stature), and age. In all the three final models, body mass resulted as the best predictor, and this is not entirely surprising given that approximately 90% of the soccer players’ body mass was determined by FFM components. Similarly, the resistance index (calculated as stature^2^/resistance) appeared as the second predictor variable in all the developed models and this is the result of the well-known conductive properties of soft tissues [5]. Using the new predictive models, BIA-based estimation of FFM, LST, and ALST were highly correlated (R^2^ ranged from 0.83 to 0.91) with the reference values obtained from DXA. Similarly, the generalized equations [32, 33] explained from 88 to 91% of the variance observed in the reference data. In comparison with the generalized models, the standard error of estimation was lower for all the new equations, ranging from 1.38 to 1.73 kg. A moderate to substantial strength of agreement between the methods was observed in estimating FFM components using the new equations (CCC > 0.90), while a poor agreement was found between the methods when former generalized equations were applied. Concerning agreement analysis, a trend was verified between the mean and the difference of methods with large limits of agreement for all the selected athletic equations [32, 33]. The underestimation of FFM components from generalized equations was already showed when equations for the normal population were applied on athletes [22]. This could be due to different body composition features, such as lower FFM values that discriminate the general population with respect to the athletic one [42]. Similarly, the inclusion of athletes from different sports in the previous development studies [32, 33], even though they may have satisfied an initial request for predictive equations to be applied in the context of sport [20], can result in a loss of accuracy when athletes of specific sports are evaluated. The athletes included in the development of the previous predictive models were actively involved in different sport disciplines such as basketball, handball, swimming, triathlon, judo, pentathlon, athletics, tennis, rowing, sailing, karate/taekwondo, boxing, hokey, climbing, rugby, soccer, fencing, motorsports, power lifting, padel, futsal, trail running, korfball, surfing, and gymnastics. These groups of athletes presented average values of 47.7 kg and 29.4 kg for FFM [33] and ALST [32], respectively. In contrast, the participants of the present study had higher FFM and ALST values, which are in line with previous studies on soccer players [29, 43]. On these bases, bias in the estimation of FFM components may occur when assessing group of subjects with different body composition features than the ones on which predictive BIA-based models are developed.

Bioelectrical characteristics of the soccer players involved in the present study where initially compared with the reference data provided on elite soccer players in 2014 [29]. This comparison showed that body composition features have not gone through a changing trend in the last decade, since individual, as well as mean bioimpedance vectors of the soccer players fell within the 50% reference tolerance ellipse. Considering the individual vectors, they were distributed within the BIVA soccer-specific references [29], with some vectors positioned out of the 95% tolerance ellipse. According to the BIVA basics, such as vectors may represent subjects with a lower fluid content with respect to the average soccer population. Since a low total body water may depend by a low body mass, long vectors should not be always related to a hypohydration state. Indeed, the characteristic and innovative aspect of BIVA is that it provides soft tissue classification (under, normal, and over) and ranking (more or less than before intervention), comparing the position of an individual vector to a reference population [5]. Somatotype was also assessed among the participants and three different morphologies (i.e., balanced mesomorph, ectomorphic mesomorph, and mesomorph-ectomorph) were identified according to the Heath and Carter method [34]. In line with previous studies, anthropometric dimensions of elite soccer players revealed a profile characterized by high musculoskeletal as well as loglinear components, with a low percentage of body fat [25, 44]. The attempt to include somatotype into the new predictive models had the aim of addressing any possible differences in body composition due to the role. However, morphologic characteristics appeared to be independent of the players positions and this may reflect the profile of the new millennium soccer player, where body dimensions can be similar among roles. Indeed, recent studies highlighted how body composition differs according to gender, competitive levels, and age groups rather than the playing position in soccer players [44, 45]. Even if a somatotype-related code was not included among the independent variables of the new predictive models, the interaction of body morphology with body composition should be tested in other sports [18, 46, 47]. This could be the case with rugby defenders where high body fat could be useful during tackles or with the role of libero in volleyball or playmaker in basketball where a shorter stature might be found [47–49].

From a practical point of view, the assessment of FFM components with generalized athletic equations should be interpreted with caution for individual estimations of FFM and ALST. Indeed, the use of generalized athletic equations on soccer players with lower FFM values resulted in overestimations (lower 95% LoA = −5.6 kg and −11.3 kg, for FFM and ALST respectively), whereas when applied on soccer players with higher values of FFM and ALST led to a systematic underestimation of the aforementioned body mass components (higher 95% LoA = 0.1 kg and −6.1 kg, for FFM and ALST respectively). Considering that during the preparatory phase athletes are commonly advised to consume protein intakes in the range of ∼2.3–3.1 g for each kg of FFM, its underestimation may result in a hypoproteic diet and in an impairment of the maintenance of the skeletal muscle structures [12]. Regarding the ALST, its reduction could be expected during transition periods and therefore closely monitored in soccer players [50]. However, although greater accuracy in ALST estimation has been achieved with the new predictive model, we are not yet aware of whether the standard error of the estimates and the observed LoA are acceptable for coaches and medical personnel for prescribing specific nutritional and training strategies. Furthermore, among the body composition parameters that can be quantified with BIA there is also the fat mass, a component that negatively affects the soccer performance [44] and that is scrupulously monitored during the transaction periods [51]. Professional soccer players present fat mass values ranging from 11 to 14%, depending on the assessment method [19]. The findings of the present study showed that a high accuracy in the FFM estimation may allow to calculate the fat mass as the difference between body mass and FFM without possible overestimations. Indeed, the use of a generalized equation [33] resulted in an underestimation of FFM and then in an overestimation of the fat mass in the soccer players. As already mentioned, smaller parts of the FFM such as LST and ALST are closely related to skeletal muscle components and particularly informative of strength and power expression in elite soccer players [43]. To our knowledge, there are currently no specific reference values ​​for elite soccer players, but with respect to body fat, lean soft tissues are more sensitive to variations due to detraining periods where a rapid decrease can be experimented [50]. Therefore, valid estimations of LST and ALST can be useful for monitor the response of training regimes across the different phases of the season or during the return-to-play after an injury [52]. With that said, the use of the new soccer-specific equations should be encouraged when assessing body composition in soccer players with BIA.

Despite the positive results obtained in the present investigation, some limitations must be acknowledged. First, although DXA represents one of the most accurate technique for assessing lean soft tissues it is not considered the state-of-the-art method for evaluating fat and FFM, for which it should be employed in conjunction with air plethysmography and dilution techniques according to a four-compartment body composition model [14]. Second, this cross-sectional design prevents from assessing predicted FFM components over time. Hence, future longitudinal and interventional studies are warranted to validate the reliability of this novel equations and their use in tracking changes in body composition across a competitive season. Lastly, the present findings cannot be generalized to other sport disciplines, females, sub-elite or adolescent players, and they cannot be extended to BIA-measures obtained from different technologies or sampling frequencies due to the lack of agreement between bioelectrical impedance analysers [53, 54]. These limitations highlight the specificity of the BIA equations concerning each technology and population. Conversely, the applicability of the new equations may extend to elite soccer players worldwide, given that the participants in this study originated from diverse territories, reflecting the typical composition of high-level soccer teams.

## Conclusions

This investigation shows that generalized BIA-based equations for athletes result in an underestimation of FFM components when applied to elite soccer players. The new developed and validated soccer-specific predictive equations are the first to provide practitioners the possibility to achieve high accuracy using BIA in the context of soccer. This allows to manage training and nutritional strategies aiming to enhance body composition and soccer performance, while maintaining the portability of the BIA.

## Data Availability

The datasets used and/or analysed during the current study are available from the corresponding author on reasonable request.
